# Aberrantly Glycosylated IgA1 as a Factor in the Pathogenesis of IgA Nephropathy

**DOI:** 10.1155/2011/470803

**Published:** 2011-01-24

**Authors:** Mototsugu Tanaka, George Seki, Tomonosuke Someya, Michio Nagata, Toshiro Fujita

**Affiliations:** ^1^Department of Internal Medicine, Faculty of Medicine, University of Tokyo, 7-3-1 Hongo, Bunkyo-ku, Tokyo 113-0033, Japan; ^2^Department of Pediatrics, Juntendo University School of Medicine, Tokyo 113-8431, Japan; ^3^Molecular Pathology, Biomolecular and Integrated Medical Sciences, Graduate School of Comprehensive Human Sciences, University of Tsukuba, Ibaraki 305-8575, Japan

## Abstract

Predominant or codominant immunoglobulin (Ig) A deposition in the glomerular mesangium characterizes IgA nephropathy (IgAN). Accumulated glomerular IgA is limited to the IgA1 subclass and usually galactose-deficient. This underglycosylated IgA may play an important role in the pathogenesis of IgAN. Recently, antibodies against galactose-deficient IgA1 were found to be well associated with the development of IgAN. Several therapeutic strategies based on corticosteroids or other immunosuppressive agents have been shown to at least partially suppress the progression of IgAN. On the other hand, several case reports of kidney transplantation or acquired IgA deficiency uncovered a remarkable ability of human kidney to remove mesangial IgA deposition, resulting in the long-term stabilization of kidney function. Continuous exposure to circulating immune complexes containing aberrantly glycosylated IgA1 and sequential immune response seems to be essential in the disease progression of IgAN. Removal of mesangial IgA deposition may be a challenging, but fundamental approach in the treatment of IgAN.

## 1. Introduction

Since IgA nephropathy (IgAN) was firstly described by Berger and Hinglais in 1968 [1], IgAN is regarded one of the most common forms of glomerulonephritis worldwide [2]. Because 30 to 40% of IgAN patients reach end-stage kidney disease within 20 years [2, 3], it is critical to clarify the true pathogenesis. Clinical onset of IgAN is probably more common in the second and third decades of life [4–7]. This disease is more common in men, but reported male-female ratios were ranging from less than 2 : 1 in Japan to as high as 6 : 1 in the United States and northern Europe. Although IgAN occurs in all ethnic groups, the reason why whites and Asians are more prone to IgAN than are blacks remains unclear [8, 9]. Approximately 50% of newly diagnosed glomerulonephritis in Japan is due to IgAN, though much lower rates are reported in the United States and Western Europe [3]. Although there seems to be true racial differences due to a genetic predisposition to IgAN, the differences in kidney biopsy practices may also reflect these disparities [10].

IgAN is characterized by predominant or codominant IgA deposits in the glomerular mesangium, but many other diseases including Henoch-Schönlein purpura nephritis (HSPN) are also associated with glomerular IgA deposits [10]. Now, IgAN and HSPN are recognized to be related diseases since both can be encountered consecutively in the same patient, are found in identical twins, and bear identical pathological and biological abnormalities [10]. Recent studies strongly suggest that aberrant glycosylation of the *O*-linked glycans in IgA1 hinge region is an important common pathogenic factor contributing to the development of IgAN [10–14] and HSPN [15, 16]. Galactose-deficient IgA1 is recognized by naturally occurring IgG or IgA1 antibodies resulting in formation of immune complexes [13]. The circulating immune complexes thus formed have a tendency to accumulate in glomerular mesangium and are considered as a main cause of glomerular injury, though the entire pathogenesis of these diseases remains still unresolved.

Herein, we review the recent development of basic and clinical investigations in IgAN and also refer to an approach aiming at IgA removal as a future therapeutic potential.

## 2. Pathogenesis of Aberrantly Glycosylated IgA1 in IgAN

IgAN is the disease of glomerular IgA deposition. Glomerular deposition of IgA requires several potential mechanisms including increased production of IgA in bone marrow, decreased clearance of IgA, and binding of IgA to mesangial cells [10]; however, the true pathogenesis has not been clearly defined. Recent data suggest that aberrant glycosylation of the *O*-linked glycans in IgA1 hinge region is a key factor in the development of IgAN. Because deposited IgA in the mesangium is restricted to IgA1 subclass [11, 12], researchers had so far attempted to clarify the qualitative or quantitative aspects of IgA1 abnormalities in IgAN.

Increased production of IgA1 in the bone marrow is thought to be responsible for the elevation of serum IgA1 [19, 20]; however, IgA-producing myeloma is usually unaccompanied by IgAN, and only the patients with aberrantly glycosylated IgA1 develop glomerulonephritis in cases with IgA1 multiple myeloma [21, 22]. Therefore, the qualitative abnormality of IgA molecules is now recognized as a fundamental cause of developing IgAN.

Unlike IgA2 and other immunoglobulins, IgA1 has a unique structure of multiple *O*-glycosylation sites in its hinge region. Although *O*-linked glycans in the hinge region in healthy individuals are composed of N-acetylgalactosamine with *β*1,3 linked galactose, a majority of the IgA1 in the glomerular deposits in patients with IgAN is galactose-deficient. This aberrantly glycosylated IgA1 is also detected in sera and in circulating immune complexes in IgAN patients, while it is rarely found in healthy controls [10–14]. The similar abnormality in IgA1 glycosylation has been also confirmed in HSPN [15, 16].

The synthesis of the *O*-glycans in human IgA1 hinge regions is initiated by the addition of N-acetylgalactosamine (GalNAc) to serine or threonine residues through the activity of UDP-N-acetyl-alpha-D-galactosamine (especially, pp-GalNAc-T2) [23]. The *O*-glycan chain is then extended by sequential attachment of galactose and sialic acid residues to GalNAc. Galactose is attached to GalNAc by core 1 *β*1,3-galactosyltransferase (C1GalT1) coordinated with its chaperone of core-1-*β*3-Gal-T specific molecular chaperone (Cosmc) [24–26], and also sialic acid (N-acetylneuraminic acid, NeuNAc) is attached to GalNAc by *α*2,6-GalNAc-sialyltransferase2 (ST6GalNAc2) [17]. Therefore, one can hypothesize that the imbalance in the activities of C1GalT1/Cosmc and ST6GalNAc2 [10, 18] can contribute to the undergalactosylation of IgA1 in IgAN. Results from a recent *in vitro* study by Suzuki et al. supported this hypothesis [27]. They demonstrated the C1GalT1 activity was significantly lower, while the ST6GalNAc2 activity was significantly higher in EBV-immortalized IgA-secreting lymphocytes from patients with IgAN. Indeed, a more recent analysis showed that the specific haplotype combinations in C1GalT1 and ST6GalNAc2 were well associated with a predisposition for IgAN and renal outcomes [28]. However, it remains controversial whether the undergalactosylation of IgA1 is a direct consequence of functional changes in C1GALT1/Cosmc or ST6GalNAc2 activities [14]. In addition to the two glycosyltransferase genes, various genes such as 6.5-cM region, *IGAN1*, in 6q22-23, and another region in chromosome 2q36 have been identified as disease-related factors in IgAN. However, the exact roles of these genetic regions in the pathogenesis of IgAN still remain to be clarified [29, 30].

Higher levels of serum galactose-deficient IgA1 in patients with IgAN and their relatives than in healthy controls rather support a genetic predisposition of aberrant IgA glycosylation [31]. The observed inheritance pattern of galactose-deficient IgA1 levels might suggest an autosomal dominant transmission. Because relatives with higher serum levels of aberrantly glycosylated IgA1 were mostly asymptomatic, additional cofactors other than the aberrant IgA glycosylation may be required for the development of IgAN [31].

While galactose-deficient IgA1 alone is insufficient to produce IgAN, the IgG antibodies and IgA1 antibodies may also play an important role in the pathogenesis of IgAN by forming circulating immune complexes containing aberrantly glycosylated IgA1 [13]. Galactose-deficient IgA1 molecules are recognized by glycan-specific IgG or IgA1 antibodies, and IgA1-containing immune complexes are formed [32, 33]. Then, galactose-deficient IgA1, as a component of the large immune complexes, may easily escape hepatic catabolism and have a longer life-span than normal IgA1 [34]. Deposited IgA1 may, in turn, induce many kinds of inflammatory mediators. Cytokines and growth factors, such as interleukin-6 [35, 36], transforming growth factor *β*1 [37–39], and platelet-derived growth factor [40] in sera of patients with IgAN were associated with mesangial proliferation or tubulointerstitial fibrosis. 

**Figure 1:**
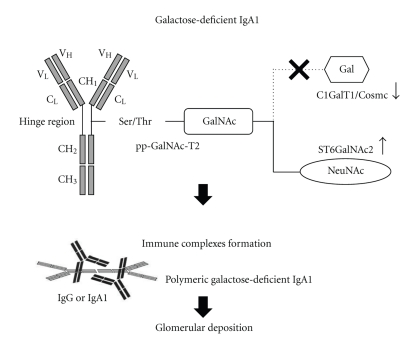
The galactose-deficient IgA1 molecule and the immune complexes formation in the pathogenesis of IgAN. IgA1 has characteristic hinge regions between the CH_1_ and CH_2_ domains (CH, the constant regions of the heavy chain), which contain at least six serine (Ser) or threonine (Thr) residues as *O*-linked glycosylation sites. In the first step, the enzyme polypeptide N-acetylgalactosaminyltransferase (pp-GalNAc-T2) facilitates the attachment of N-acetylgalactosamine (GalNAc) to these residues. In the second step, the IgA1 glycosylation is extended in two ways. One is the binding of N-acetylneuraminic acid (NeuNAc) to GalNAc through the action of the enzyme N-acetylgalactosamine-specific *α*2,6-sialyltransferas (ST6GalNAc2) [17]. The other is galactose (Gal) connection to GalNAc through the enzyme core 1 *β*1,3 galactosyltransferase (C1GalT1) and core-1-*β*3-Gal-T specific molecular chaperone (Cosmc) [18]. The imbalance between those activities may promote galactose-deficient IgA1 production [10, 18]. Galactose-deficient IgA1 is aggregated with antiglycan IgG or IgA1 antibodies, resulting in formation of immune complexes [13], which may escape the normal catabolism in liver and also accumulate with high affinity in glomerular mesangium.

These mesangial cells proliferation and glomerular injury may be induced by circulating immune complexes, in particular, containing aberrantly glycosylated IgA1. A recent *in vitro* study demonstrated that the circulating immune complexes containing galactose-deficient IgA1 prepared from sera of IgAN patients stimulated mesangial cell proliferation more efficiently than uncomplexed IgA1 or immune complexes prepared from healthy control subjects [13]. This stimulatory activity was lost in fractions devoid of IgA1. Indeed, circulating immune complexes containing higher levels of galactose-deficient IgA1 enhanced mesangial cell proliferation more efficiently than complexes with lower levels of galactose-deficient IgA1 [13]. Another recent *in vitro* study, using subcloned EBV-immortalized B cells from IgAN patients and healthy controls, revealed that the elevation in serum levels of antibodies against galactose-deficient IgA1 is well associated with the development of IgAN [41]. These findings strongly suggest that circulating immune complexes containing aberrantly glycosylated IgA1, by accumulating in glomerular mesangium in patients with IgAN, may play pivotal roles in the development and progression of kidney injury as shown in Figure 1. Further investigations about the roles of these antibodies are expected to provide new insights into the pathogenesis of IgAN.

Although alterations in IgA1 glycosylation certainly play an important role in the progression of kidney disease in IgAN, serum levels of galactose-deficient IgA1 are not necessarily elevated in a significant proportion of patients with IgAN [42, 43]. Thus, it should be emphasized that the abnormality in IgA1 glycosylation may not be the only mechanism underlying the development of IgAN.

## 3. Recent Clinical Trials in Treatment for IgAN

A randomized controlled trial (RCT) of treatment for IgAN reported by Pozzi et al. showed a remarkable efficacy of 6-month corticosteroids therapy (intravenous methylprednisolone 1 g for 3 days at months 1, 3, and 5, followed by oral prednisone 0.5 mg/kg/alternate-day for 6 months) [44]. The follow-up analysis of this study showed that the proportion of patients who reached 50% increase in serum creatinine was significantly lower in the corticosteroids group than in the control group at 10 years after the treatment [45]. The beneficial effects of angiotensin-converting enzyme inhibitor (ACEi) in the treatment for IgAN were reported in another RCT by Praga et al. [46]. In this study, while blood pressure control was similar between the two groups, the patients who reached the same primary endpoint were less in the ACEi group than in the control group. Recently, RCTs by Manno et al. [47] and Lv et al. [48] showed the additive benefits of corticosteroids on the ACEi-based therapy. Kidney function, during the each follow-up period, was more preserved in the combination therapy group than that in the control group. Decline of estimated glomerular filtration rate (eGFR) was slower, and more strict control of proteinuria was achieved in the combination therapy group than in the ACEi monotherapy group. However, a recent critical review pointed out that it still remained controversial whether corticosteroids should be administered in IgAN patients who were already treated with maximum recommended dose of ACEi [49]. Indeed, more aggressive immunosuppressive therapy should be undertaken carefully. A recent RCT failed to demonstrate the additional benefit of low-dose azathioprine adding to corticosteroids for 6 months [50]. Although adverse side effects were more frequent in the combination therapy group, reduction of proteinuria and 5-year cumulative kidney survival were comparable in the combination therapy group and the corticosteroids monotherapy group. Tang et al. recently reported the efficacy of mycophenolate mofetil (MMF) in the treatment of IgAN [51]. In that study, 40 IgAN patients with persistent proteinuria (>1 g/day) despite the conventional treatment with renin-angiotensin system inhibitors were randomized to the immunosuppression group receiving MMF for 6 months or the control group receiving the conventional treatment alone. They concluded that MMF provided partial remission of proteinuria in the first 2 years and better kidney survival at 6 years after the treatment. However, the observational periods of these and other studies in IgAN, published until the present time, were relatively short compared to the nature of the slow disease progression of IgAN [44–55]. Thus, it is not fully established whether these beneficial effects of immunosuppressive therapies can result in the better prognosis after the long-term follow-up period. Moreover, it has not been clear whether such immunosuppressive therapies are effective even in patients with severe IgAN, because most of the previous clinical trials were conducted on IgAN patients with mild to moderate kidney insufficiency.

## 4. Therapeutic Approach Aiming at IgA Removal

Human kidney has a considerable self-purification ability to remove IgA deposits from glomeruli, because the previous reports showed that transplanted kidneys from donors with subclinical IgAN into recipients without IgAN rapidly cleared the glomerular deposits [56, 57]. This ability may be altered in IgAN patients. Several studies, focusing on IgA receptors, showed that IgA1-containing circulating immune complexes from IgAN patients bind to mesangial cells with higher affinity than complexes from healthy controls [58, 59].

Based on the clinical experiences in kidney transplantation described above, it is also suggested that the continuous exposure to circulating immune complexes, containing aberrantly glycosylated IgA1, and sequential immune responses may be essential for the disease progression of IgAN. Thus, the fundamental treatment of IgAN seems to block these processes.

Recently, Lamm et al. reported a novel therapeutic potential for IgAN [60]. They demonstrated the ability of the bacterial IgA protease to remove glomerular IgA immune complexes in a mouse model of IgAN. Because of the absence of IgA1 with its hinge region in such animals, they designed a passive mouse model of IgAN by injecting immune complexes containing human IgA1. After the systemically administration of the IgA protease derived from* Haemophilus influenza*, the mesangial IgA immunofluorescence intensities were significantly reduced. This treatment strategy, targeting the removal of mesangial IgA1 deposits, is novel and interesting; however, we have to overcome some limitations of this study for clinical application in human IgAN. First, there are some differences in IgA glycosylation, IgA clearance, and control of IgA synthesis between rodents and humans. Second, the deposition and the removal of glomerular IgA-containing immune complexes in the mice model are too rapid compared to the much slower processes in human IgAN. Moreover, the circulating load of IgA1 is substantial and may well consume the protease before it has any effect in the kidney. Furthermore, circulating neutralizing antibody may appear to lessen the impact of such a therapeutic approach. Finally, it is unknown whether the similar approach is safe and possible in human IgAN at present. Further studies are required to investigate how efficiently the IgA protease digests circulating immune complexes and deposited complexes.

**Figure 2:**
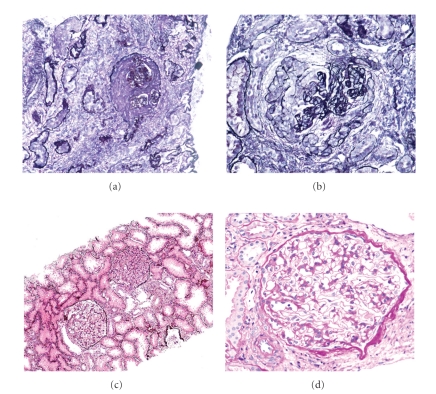
Repeated kidney biopsies in an HSPN patient with acquired IgA deficiency. (a, b) The first kidney biopsy (performed at 7 years old) showing necrotizing crescentic glomerulonephritis with advanced glomerulosclerosis and severe tubulointerstitial nephritis, which was compatible with HSPN grade 5b. (c, d) The fourth kidney biopsy (performed at 21 years old) showing minor glomerular abnormalities. Kidney biopsy samples were stained with periodic acid methenamine silver (a, b, and c), and periodic acid Schiff (d), respectively. Original magnifications were ×10 (a, c) and ×40 (b, d), respectively.

We have recently documented a case of HSPN with acquired IgA deficiency [61], which may give us some clinical hints about the efficacy of IgA removal. The patient was initially diagnosed as the severest form of HSPN (grade 5b), according to the criteria of International Study of Kidney Disease in Children [62] at 7 years old. During the immunosuppressive therapy, the patient developed virus associated hemophagocytic syndrome. Interestingly, initially elevated serum levels of IgA severely decreased after those events and remained undetectable until the present time. Although her original clinicopathological findings rather suggested a poor prognosis [63], periodic examination, and repeated kidney biopsies performed over a period of 14 years showed an excellent clinical course consisting of the normalization of kidney function, the marked reduction in protenuria, and the remarkable histological improvement as shown in Figure 2. No serious adverse clinical symptoms related to IgA deficiency were observed.

Unfortunately, we do not know whether this patient had the aberrantly glycosylated IgA1. However, our case suggests that acquired IgA deficiency works favorably in the resolution of severe HSPN.

## 5. Conclusion

Galactose-deficient glycosylated IgA1 seems to be a key factor in the pathogenesis of IgAN. Based on the recent studies, continuous exposure to circulating immune complexes containing aberrantly glycosylated IgA1 may be essential in the disease progression of IgAN. While several clinical trials based on the immunosuppressive agents reported promising effects, it remains unclear whether such therapies really improve the long-term prognosis of IgAN.
